# National policies for delivering tuberculosis, HIV and hepatitis B and C virus infection services for refugees and migrants among Member States of the WHO European Region

**DOI:** 10.1093/jtm/taac136

**Published:** 2022-11-25

**Authors:** Rebecca F Baggaley, Joshua Nazareth, Pip Divall, Daniel Pan, Christopher A Martin, Mikhail Volik, Nicole S Seguy, Askar Yedilbayev, Marge Reinap, Elena Vovc, Antons Mozalevskis, Andrei Dadu, Elisabeth Waagensen, Krista Kruja, Tyrone Reden Sy, Laura Nellums, Manish Pareek

**Affiliations:** Department of Population Health Sciences, University of Leicester, Leicester LE1 7RH, UK; National Institute for Health and Care Research (NIHR) Leicester Biomedical Research Centre, LE1 7RH, UK; Department of Respiratory Sciences, University of Leicester, Leicester LE1 9HN, UK; University Hospitals of Leicester NHS Trust, Leicester LE1 5WW, UK; University Hospitals of Leicester NHS Trust, Leicester LE1 5WW, UK; National Institute for Health and Care Research (NIHR) Leicester Biomedical Research Centre, LE1 7RH, UK; Department of Respiratory Sciences, University of Leicester, Leicester LE1 9HN, UK; University Hospitals of Leicester NHS Trust, Leicester LE1 5WW, UK; Li Ka Shing Centre for Health Information and Discovery, Oxford Big Data Institute, University of Oxford, Oxford OX3 7LF, UK; Department of Respiratory Sciences, University of Leicester, Leicester LE1 9HN, UK; University Hospitals of Leicester NHS Trust, Leicester LE1 5WW, UK; Division of Country Health Programmes, WHO Regional Office for Europe, 2100 Copenhagen Ø, Denmark; Division of Country Health Programmes, WHO Regional Office for Europe, 2100 Copenhagen Ø, Denmark; Division of Country Health Programmes, WHO Regional Office for Europe, 2100 Copenhagen Ø, Denmark; Division of Country Health Policies and Systems, WHO Regional Office for Europe, 2100 Copenhagen Ø, Denmark; Division of Country Health Programmes, WHO Regional Office for Europe, 2100 Copenhagen Ø, Denmark; Division of Country Health Programmes, WHO Regional Office for Europe, 2100 Copenhagen Ø, Denmark; Division of Country Health Programmes, WHO Regional Office for Europe, 2100 Copenhagen Ø, Denmark; Division of Country Support, Emergency Preparedness and Response (CSE), WHO Regional Office for Europe, 2100 Copenhagen Ø, Denmark; Division of Country Health Policies and Systems, WHO Regional Office for Europe, 2100 Copenhagen Ø, Denmark; Division of Country Health Policies and Systems, WHO Regional Office for Europe, 2100 Copenhagen Ø, Denmark; Nottingham Centre for Epidemiology and Public Health, Lifespan and Population Health, University of Nottingham, Nottingham NG7 2UH, UK; National Institute for Health and Care Research (NIHR) Leicester Biomedical Research Centre, LE1 7RH, UK; Department of Respiratory Sciences, University of Leicester, Leicester LE1 9HN, UK; University Hospitals of Leicester NHS Trust, Leicester LE1 5WW, UK

**Keywords:** Refugees; migrants, infections, tuberculosis, HIV, viral hepatitis, Europe

## Abstract

**Background/objective:**

Refugees and migrants to the World Health Organization (WHO) European Region are disproportionately affected by infections, including tuberculosis (TB), human immunodeficiency virus (HIV) and hepatitis B and C (HBV/HCV) compared with the host population. There are inequities in the accessibility and quality of health services available to refugees and migrants in the Region. This has consequences for health outcomes and will ultimately impact the ability to meet Regional infection elimination targets.

**Methods:**

We reviewed academic and grey literature to identify national policies and guidelines for TB/HIV/HBV/HCV specific to refugees and migrants in the Member States of the WHO European Region and to identify: (i) evidence informing policy and (ii) barriers and facilitators to policy implementation.

**Results:**

Relatively few primary national policy/guideline documents were identified which related to refugees and migrants and TB [14 of 53 Member States (26%), HIV (*n* = 15, 28%) and HBV/HCV (*n* = 3, 6%)], which often did not align with the WHO recommendations, and for some countries, violated refugees' and migrants’ human rights. We found extreme heterogeneity in the implementation of the WHO- and European Centre for Disease Prevention and Control (ECDC)-advocated policies and recommendations on the prevention, diagnosis, treatment and care of TB/HIV/HBV/HCV infection among migrants across the Member States of the WHO European Region.

There is great heterogeneity in implementation of WHO- and ECDC-advocated policies on the prevention, diagnosis, treatment and care of TB/HIV/HBV/HCV infection in refugees and migrants across the Member States in the Region.

**Conclusion:**

More transparent and accessible reporting of national policies and guidelines are required, together with the evidence base upon which these policy decisions are based. Political engagement is essential to drive the changes in national legislation to ensure equitable and universal access to the diagnosis and care for infectious diseases.

## Background

Migration has increased substantially in the World Health Organization (WHO) European Region.^1^ In 2020, the United Nations Population Division estimated that almost 13% of the total population of the region were foreign-born,^2^ and since then, the conflict in Ukraine has caused millions of refugees to cross borders into neighbouring countries. The prevalence of tuberculosis (TB; active and latent), human immunodeficiency virus (HIV) infection and chronic viral hepatitis [specifically, hepatitis B virus (HBV) and hepatitis C virus (HCV) infections] may be higher in refugees and migrants. However, refugees and migrants do not normally pose a health risk to the host population.^3^^,^^4^ The reasons for the relatively increased burden of infectious diseases in some refugee and migrant populations are complex and multifactorial, but risk factors related to origin, transit and destination countries can include a high prevalence of infection, under-resourced healthcare systems, low immunization coverage, lack of accessible healthcare and poor living conditions.^5–7^ Undetected and untreated TB and HIV, HBV and HCV infections lead to poor health outcomes: a study of refugee and migrant mortality in five western European countries found a heterogeneous pattern of differences in all-cause and infectious disease-related mortality between different refugee/migrant and local-born populations, with higher mortality rates due to infectious diseases, in particular, from TB and HIV/AIDS in most refugee and migrant populations.^8^

**Table 1:** Summary of WHO and ECDC guidance and recommendations relating to TB, HIV, HBV and HCV infection and refugees and migrants to the WHO European Region

TB	HIV	HBV/HCV
Action plans, guidance, recommendations and strategies		
Action plans/strategies for TB control in WHO European Region^9–14^ Roadmap to implement TB action plan for WHO European Region 2016–20^11^: divides recommendations for TB management in refugees and migrants into three pillars which form part of the End TB Strategy: refugee and migrant-sensitive care and prevention bold intersectoral policies and systems supportive of refugees and migrants operational research WHO evidence-based guidance/recommendations for implementing the End TB Strategy^14–16^: Active TB screening: systematic screening in subpopulations with very high TB rates or very poor access to healthcare, such as some refugees and migrants residing in, or coming from, settings with a high TB prevalence^85,b^ LTBI management^c^: systematic testing and treatment of LTBI for refugees and migrants according to TB epidemiology and resource availability^16^ Either IGRA or TST should be used^d^ Global framework towards TB elimination in low-incidence countries:^21,i^ Interventions for refugees and migrants: Ensure healthcare services are accessible to all refugees and migrants, with individual follow-up Detailed surveillance including disaggregated data on refugee and migrant groups to identify those at highest risk Empower refugee and migrant communities Systematic active TB screening Systematic LTBI screening focussing on groups at high risk of exposures and of progressing to active TB Screening should observe human rights principles and safeguard against stigma, discrimination and deportation Cross-border referral systems with contact tracing and information sharing	WHO Action Plan for the Health Sector response to HIV in the WHO European Region (2017)^17^: Guiding principle: universal health coverage ensuring all PLWH can access the full range of healthcare services they need Targets covering all areas of HIV management to achieve goals of reducing HIV incidence Eliminate HIV-related discrimination Provide universal treatment^e^ WHO: ART to be initiated in all adult PLWH regardless of clinical stage and CD4 count^18^ WHO recommends use of PrEP for individuals with ≥3% risk per year of acquiring HIV^65,f^ ECDC recommendations for refugees and migrants^19^: Offer HIV screening to refugees and migrants who have lived in communities with high HIV prevalence (≥1%) If HIV positive, link to care and treatment as per clinical guidelines Offer testing for HIV to all adolescents and adult refugees and migrants at high risk for exposure to HIV ECDC priority action^20^: reduce legal and policy barriers in place for undocumented migrants to receive ART	WHO: first action plan for viral hepatitis for the WHO European Region adopted by all 53 Member States in 2016^21^ Action plan goal: eliminating viral hepatitis as a public health threat in the WHO European Region by 2030 through reducing transmission, morbidity and mortality. Global targets of 80% reduction in new chronic infections and 65% reduction in mortality from the 2015 levels. Recommendations to reach these targets that relate to refugees and migrants: Improved data are necessary both within a country’s health system to integrate with broader HIS and in cross-border systems to enable better continuity of care 50% of all people living with chronic hepatitis B and C to be diagnosed by 2020 through improved testing and screening Prevention of mother-to-child transmission through screening pregnant women from countries not implementing universal HBV vaccination and access to post-exposure prophylaxis for newborns, where needed Reducing sexual transmission: ensuring access to dedicated sexual and reproductive health services Strengthening human resources using community-based organizations and peer-support workers for vulnerable populations such as refugees and migrants ECDC recommendations for refugees and migrants^19^: HBV screening and treatment to be offered to refugees and migrants from intermediate- and high-prevalence countries (≥2 and ≥5% HBsAg positivity, respectively)^h^ HBV vaccination to be offered to all refugee and migrant children and adolescents from intermediate- and high-prevalence countries who do not have evidence of vaccination or immunity HCV antibody screening to be offered to refugee and migrant populations from HCV-endemic countries (≥2% positivity) Refugees and migrants with anti-HCV antibodies to undergo RNA testing and those testing positive to be linked to care and treatment
ECDC recommendations for refugees and migrants^19^: Offer active TB screening (CXR) soon after arrival for refugees and migrants from high TB incidence countries. Offer LTBI screening (TST/IGRA) soon after arrival for all refugee and migrant populations from high TB incidence countries and link to care and treatment where indicated ECDC recommendations for vulnerable populations, including refugees and migrants:^18,a^ Outreach teams and mobile units for testing and treatment Involvement of key partners to support patients with their treatment and find contacts Directly observed and video-observed treatment Reminders to improve patient attendance and medication uptake Integration of services Promoting awareness and education to tackle stigma and misconceptions		
Discrimination		
Refugees and migrants are protected under international law from blanket restrictions on entry, stay and residence based solely on their TB status.^22^ WHO additional guidance to ensure that sound ethics underpin implementation of the End TB Strategy: screening and testing of refugees and migrants may only be justified with the objective to provide adequate medical care and never to discriminate^14^	WHO, UN and UNAIDS strongly advise against restricting movement of PLWH.^17^^,^^18^^,^^23^ Such restrictions are discriminatory, unjustified and not supported by public health evidence^62,g^ ECDC and WHO strongly advise against mandatory HIV testing of refugees and migrants, although ECDC recommends that refugees and migrants from countries with a high HIV prevalence (≥1%) should be offered an HIV test.^17^^,^^19^ HIV testing should be voluntary and confidential, with informed consent	

ART, antiretroviral therapy.

^a^Further details of the ECDC recommendations^9^ are summarized in Table 2, WHO Health Evidence Network synthesis report 74.^24^

^b^Based on very low-quality evidence due to lack of available data. No threshold for high-prevalence is provided; WHO recommends that it must be adapted to the local context and may also change over time.

^c^In high- or upper-middle-income countries with a low TB burden (incidence < 100 cases/100 000 population per year).

^d^Conditional recommendation, based on low to very low quality of evidence, Appendix Table A6.

^e^See Appendix Box A1, Appendix, for details. The Plan advocates for interventions for key populations (including refugees and migrants) which are tailored to the local context, capacity and resources and ensures that services are relevant, acceptable and accessible. One fast-track action is for improved information with the need to expand cross-border sharing of information to ensure continuity of care of mobile populations, including migrants.

^f^The main obstacles are inability to identify high-risk subgroups of refugees and migrants who would benefit from PrEP and the lack of refugee and migrant-specific services to provide it.^25^

^g^These policies emphasize the discrimination and stigmatization faced by PLWH, which may cause them to conceal their diagnosis, thereby preventing them accessing the healthcare services they require.^26^

^h^Different thresholds of HBsAg positivity were used in the guidelines and policies identified in the search to categorize countries as of low, intermediate and high prevalence.

^i^Framework authored by multiple WHO employees, including corresponding author. Further details are summarized in Table 1, WHO Health Evidence Network synthesis report 74.^24^

**Table 2:** Summary of WHO European Region countries with identified policies or guidelines^a^ relating to TB, HIV, HBV and HCV infection and refugees and migrants, and the alignment of these with WHO and ECDC guidance

TB	HIV	HBV/HCV
Belgium	Azerbaijan	France
Finland	Bosnia and Herzegovina	Italy
France	Bulgaria	UK
Germany	Cyprus	
Greece	Czechia	
Ireland	France	
Italy	Greece	
The Netherlands	Israel	
Norway	Kazakhstan	
Russian Federation	Kyrgyzstan	
Spain	Luxembourg	
Sweden	Russian Federation	
Switzerland	Turkmenistan	
UK	Ukraine	
	UK	
Of all (*n* = 53) WHO European Region countries: 14 (26%) have any identified TB guidance/policies 8 (15%) have guidance/policies in alignment with the WHO/ECDC	Of all (*n* = 53) WHO European Region countries: 15 (28%) have any identified HIV guidance/policies 3 (6%) have guidance/policies in alignment with the WHO/ECDC	Of all (*n* = 53) WHO European Region countries: 3 (6%) have any identified HBV/HCV guidance/policies 2 (4%) have guidance/policies in alignment with the WHO/ECDC

All countries listed are identified as having some refugee/migrant-specific policies in place (either national policy/guidance documents identified, or secondary reporting of national policies through articles included in our review). Green—policies/guidance broadly in alignment with WHO/ECDC guidance, as summarized in Table 1; amber—insufficient information on policies/guidance to determine alignment, or in partial alignment only; red—policies/guidance in contradiction with WHO/ECDC (in all cases, this related to discrimination against PLWH: restriction of movement and/or confidential and voluntary testing).

^a^‘Relevant’ is defined as any guidance or policy relating to the relevant infectious disease and refugees and migrants. Guidance and policy documents are included if they were identified by this review or were cited by other reviewed publications.

**Table 3:** Barriers and facilitators affecting the implementation of TB, HIV and hepatitis B and C services to refugees and migrants, stratified by systems level

Systems level	Barriers	Facilitators
Macro		
Policy	Restrictive immigration and health policies Data sharing Charging Complex entitlement regulations	Political commitment Integration into government plans/strategies Separation of health and legal systems Universal, affordable healthcare
Transnational	Lack of cross-border collaboration Poor quality data	Robust data collection, monitoring and evaluation Patient confidentiality and data protection Transnational continuity of care Cross-border collaboration and policy Cross-sectoral initiatives
Population	High mobility/dispersal	Voluntary testing
Meso		
Community	Lack of community support Stigma Racism Multiple discriminations Lower social status	Clear health information and messaging Involvement and engagement of affected communities Peer-support/community champions Public education and awareness-raising
Service	Structural discrimination Fragmentation/lack of joined-up care Knowledge and attitudes of health professionals Lack of provider awareness of entitlements Limited opening hours Inconsistencies in testing, treatment and charging across services Distance to services Time constraints of services^a^	Multi-disease services Locally tailored interventions Availability of community-based services Health insurance/free at the point of care Training to ensure services are inclusive, diversity sensitive and culturally relevant Health service flexibility Broad range of screening and treatment services
Micro		
Structural	Limited knowledge of health and social care services, entitlements or protections Insecure legal status Fear Lack of trust	Patient involvement in healthcare decisions and delivery Patient and community ownership Clear patient pathways Holistic approach/improving overall health and health-seeking behaviour Patient-centred approaches Awareness of rights and entitlements
Sociocultural	Language Religion Health belief models Social and emotional isolation Low self-perceived risk	Social support networks Access to interpreting/translation services Culturally relevant pre- and post-testing counselling
Socio-economic	Social and economic insecurity from high-risk living conditions and poor working conditions Lack of health insurance Insecure housing Limited transportation	Convenient, patient-friendly outreach settings

Table adapted from Table 8, WHO Health Evidence Network synthesis report 74.^24^

^a^Resulting from limited opening/appointment times and a high demand for services.

Although TB incidence in the WHO European Region is decreasing, a large proportion of cases are detected in refugees and migrants but with significant heterogeneity between Member States.^27^ TB patients of foreign origin represent 33% of cases in the European Union (EU)/European Economic Area (EEA) countries but only 4·3% in non-EU/-EEA countries.^27^ Latent TB infection (LTBI) is an asymptomatic, non-infectious form of TB, but active TB can potentially develop, leading to symptoms and potential transmission. Although the risk of reactivation in refugees and migrants with untreated LTBI is unclear, one study of untreated refugees and migrants from high-TB-incidence countries to the UK with a positive tuberculin skin test (TST) reported that 16·3% progressed to active TB within 15 years of arrival.^28^

In 2020, in the EU/EEA, refugees and migrants accounted for 36% of the total HIV diagnoses and 44% of those with known information on the region of origin.^29^

There is considerable heterogeneity in both HBV and HCV prevalence among refugees and migrants.^30^^,^^31^ Most cases of chronic HBV infection in northern and western European countries are detected in refugees and migrants, and prevalence may be higher among refugees and asylum seekers compared with all migrants (9.6 vs 5.1%). HBV prevalence in first-generation migrants, refugees and pregnant migrants is generally higher in refugees and migrants from the south-east Asian, east European and sub-Saharan African countries, more heterogeneous in refugees and migrants from the east Mediterranean region and Latin America and generally lower in south Asian refugees and migrants. A large proportion of individuals with chronic HCV infection are asymptomatic and undiagnosed, making it difficult to estimate the true disease burden in the refugee/migrant population. Refugees and migrants from HCV-endemic countries contribute disproportionately to HCV cases (14%) in the EU/EEA and account for up to half of those living with chronic HCV in some low-HCV-prevalence EU/EEA countries,^32^ with refugees and migrants from the east European and sub-Saharan African countries having the highest reported prevalence.^31^ However, a recent analysis of electronic personal health records of recently arrived refugees and migrants in eight European countries (Bulgaria, Croatia, Cyprus, Greece, Italy, Romania, Serbia and Slovenia), in 2016–19, found that the majority of episodes of HCV were among refugees and migrants from Asia.^33^

Strategies and action plans have been developed by the European Centre for Disease Prevention and Control (ECDC), WHO and other international organizations to ensure accessible and quality healthcare for refugees and migrants which meets their health needs and human rights. The WHO Regional Office for Europe has developed action plans for refugee and migrant health and these four key infections.^9–14^^,^^34–38^

Despite the availability of such guidance, approaches to screening these infectious diseases appear to vary considerably in the WHO European Region, with no agreement on the most effective and cost-effective approaches to targeted interventions for refugees and migrants, or on those which have the best uptake and treatment outcomes.^39–42^ National guidelines and policies are critical in informing front-line clinicians of the screening practices and for reducing the regional and national heterogeneity. They also provide a picture of the current approved approach to managing communicable diseases in refugees and migrants in that country. We, therefore, sought to identify the evidence on existing national policies and guidelines for delivering effective TB/HIV/HBV/HCV infection services for refugees and migrants among Member States of the WHO European Region, to assess their alignment with WHO/ECDC guidance, and the evidence cited as informing national policies.

## Literature Search

This review was undertaken to inform a WHO Health Evidence Network synthesis report,^43^ the key findings of which are reported here. Searches of peer-reviewed literature in four English-language databases (Embase, Health Management Information Consortium, Medline and OpenGrey) from inception to present were carried out on 30 November 2020. Evidence was obtained through systematic review of the academic and grey literature in English and Russian and through the Ministry of Health websites and in consultation with the WHO TB, HIV, Hepatitis and Migration networks conducted on 30 November 2020. Search and translation of Russian publications were undertaken to ensure adequate representation of Eastern European literature. Results of the search are shown in Appendix Figure A1. Following full review and data extraction, framework analysis was conducted to identify relevant themes. Further details of the review methods can be found in the appendix.

The primary aims of our review were to analyse national policies, strategies and guidelines which have been developed and implemented to address refugee and migrant health and TB/HIV/HBV/HCV infection and to provide an overview of areas where national policies align with WHO/ECDC guidance and gaps that still need addressing. Secondary aims were to (i) describe how cost-effectiveness or national funding allocation is considered in national legislation, policies and guidelines for the implementation of TB/HIV/HBV/HCV infection services for refugees and migrants and (ii) evaluate the evidence on facilitators and barriers (at the macro-, meso- and micro-levels) to access for refugees and migrants to these health services.

For the purposes of this article, a migrant is defined as any person who is moving or has moved across an international border away from their habitual place of residence, regardless of the person’s legal status, whether the movement is voluntary or involuntary, or what the causes for the movement are.^44^^,^^45^ This is a modified version of the International Organization for Migration (IOM) definition, as our analysis focuses on transnational migration, and particularly, migration from outside the WHO European Region because this is the group of migrants at highest risk of living with HIV, or what the length of the stay is. Migrant populations are therefore extremely heterogeneous and include refugees, asylum seekers and undocumented migrants (defined according to the IOM Key Migration Terms.^46^) Since many regional datasets and reports compare foreign-born, non-EU-born and native populations or refer to immigrants, for clarity, we report these terms when describing these datasets.

Of the 268 articles included for data extraction, most were reviews of international policy. Other types included policy and guideline documents and empirical studies reporting policy analyses and surveys of reports by country experts on national policies and guidelines (primarily for the following countries Belgium, Cyprus, Denmark, France, Germany, Israel, Italy, the Netherlands, Norway, Spain, Sweden, Switzerland, Türkiye and the UK). Across the 53 Member States of the WHO European Region, only 15 primary policy/guideline documents relating to refugees and migrants and TB/HIV/HBV/HCV infection were identified: 3 on viral hepatitis,^47–49^ 3 on HIV,^50–52^ 7 on TB^53–59^ and 2 on a combination of these diseases.^60^^,^^61^

A summary of the WHO and ECDC recommendations relating to TB/HIV/HBV/HCV infection and refugees and migrants is provided in Table 1, with the alignment of national policies to these recommendations shown in Table 2 and discussed below.

### Tuberculosis

Screening for TB can focus on detecting an active TB disease or LTBI. LTBI can be detected using interferon-gamma release assays (IGRAs) or TST, while screening for TB disease typically involves a combination of symptom-based physical assessment and chest X-ray (CXR), with confirmatory bacteriological testing involving sputum culture (using smear microscopy, PCR and/or culture). Active TB and LTBI pose different challenges for screening and treatment in refugee and migrant populations. Screening practices for the two forms are highly heterogeneous across Europe (Appendix Table A1). Also, variation within the WHO European Region and substantial heterogeneity in approaches to screening was seen within countries. A 2018 survey among EU/EEA national TB programme leads undertaken by Collin *et al*. to investigate the screening practices for refugees and migrants in the EU/EEA found that screening for active TB was conducted for asylum seekers in 77% (*n* = 24) of countries and for refugees in 71% (*n* = 22) of countries.^62^ Post-entry screening of documented migrants for active TB was more common [42% (*n* = 13) of countries] than point of entry screening [32% (*n* = 10) of countries]. Pre-entry active TB screening has been implemented in the UK for long-stay visitors (>6 months) from countries with TB incidence >40 cases per 100 000 population. While there has been an increasing focus on pre-entry TB screening programmes, particularly in low-incidence host countries because they consider this to be more cost-effective, this approach only targets a specific subset of migrants (i.e. those with planned migration routes to receiving countries). Many migrants using alternative routes, such as refugees, asylum seekers and undocumented migrants would not be covered by these programmes. Pre-entry screening would also miss infections acquired en route to destination countries.^63^

In many countries, LTBI screening does not occur as commonly as active TB screening despite evidence suggesting that, in low-incidence countries, most cases of active TB in refugees and migrants result from LTBI reactivation (Appendix Table A1).^62^^,^^64^ A 2018 survey reported that 32% of countries (*n* = 10) screened refugees and migrants at the point of entry for active TB but only 20% (*n* = 6) screened refugees and migrants for LTBI.^62^ A similar pattern was found for post-entry testing [42% (*n* = 13) of countries screened for active disease, 17% (*n* = 6) for LTBI]. A second survey, of TB Network European Trials group members, found that 12 of the 22 countries responding to the survey had national LTBI screening policies.^65^ These findings highlight the heterogeneity across countries in the WHO European Region on which refugee and migrant groups are screened, what screening method is used and when screening takes place. Authors found that although a LTBI diagnosis would not alter a patient’s immigration status, refugees and migrants often did not receive the most up-to-date treatment regimens.^65^

Even where national policies exist for screening refugees and migrants for active TB and LTBI, levels of implementation vary considerably, e.g. whether implementation is the responsibility of local health authorities. For example, in the UK, an evaluation of local TB services found that the current guidance was not followed: only half of services attempted to screen refugees and migrants for LTBI and the recommended screening method was not always used.^66^^,^^67^ Limited service capacity in the UK for screening all refugees and migrants from countries with TB incidence >40 cases/100 000 population means local service providers may instead use a higher threshold to reduce the number of refugees and migrants eligible for screening.^68^ Further within-country heterogeneity of screening practices is caused by incomplete national policies. For example, in Germany, screening policies for children and pregnant women are lacking at national level, so different policies at a federal state level govern the screening decisions.^69^ Identified policy research studies also commented on the lack of standardized data collection tools, which would allow a systematic analysis of TB screening and management in refugees and migrants.

### HIV

A 2018 ECDC survey of national HIV testing policies in the 53 countries forming the WHO European Region identified only 10 countries (all in western Europe) with published guidelines that included specific recommendations for key populations, including refugees and migrants.^70^ National guidance on refugee and migrant testing for HIV is highly heterogeneous in the European Region, with marked disparities in HIV testing practices for undocumented migrants (Table 2).^70^ For those WHO European Region countries where policies relating to HIV care and treatment could be identified, many deviate from the international recommendations and standards, including policies relating to restriction of movement and deportation (further details as per country are provided in Appendix Table A2).

An ECDC technical report on HIV testing reported that only seven countries provided data on testing rates for refugees/migrants, and some of these were for specific migrant groups only.^70^ Testing uptake rates remain low. Only Greece was able to report the rates for undocumented migrants. Apart from Hungary, all the countries that were able to report testing rates among their refugee and migrant populations are in the West sub-region (not all reporting countries were named in the report). No reporting countries were able to provide data for 2017, demonstrating that, even where data are collected, monitoring is insufficiently frequent.

The Russian Federation and Turkmenistan are the 2 WHO European Region countries out of 19 countries worldwide which still deport or deny residency to HIV-positive non-nationals, who otherwise remain undocumented migrants.^71^ In the Russian Federation, work permit regulations are unclear and frequently changed, creating a persistent state of legal uncertainty for migrants, who ‘are “illegal” but tolerated’ and face multiple barriers to accessing health services.^72^^,^^73^ By contrast, many countries (including Bulgaria and Czechia) have recently followed the ECDC, UN and WHO advice on freedom of movement for people living with HIV (PLWH) and have removed travel restrictions.^26^^,^^74^^,^^75^

Finally, no guidelines were identified on the use of pre-exposure prophylaxis (PrEP) for refugees and migrants or how to identify migrants at high risk of HIV acquisition despite WHO guidance.^25^ Clear definitions of refugees and migrants at high risk for acquiring HIV are required to ensure such subpopulations have access to PrEP.

### Hepatitis B and C

Only four national policy documents were identified concerning refugees and migrants and the management of chronic HBV and HCV infection (Israel, Italy and the UK; the latter published separate documents for HBV and HCV,^76^^,^^77^  Appendix Table A3). Secondary evidence from research papers evaluating national policies through reviews and surveys provided useful insight into the extent to which existing regional guidelines have been implemented.

A 2013 survey sent to healthcare professionals in Germany, Hungary, Italy, the Netherlands, Spain and the UK found that specific guidelines on chronic HBV and HCV infection in refugees and migrants were mentioned by only 23% (HBV infection) and 14% (HCV infection) of the respondents.^78^ Screening policies for HBV infection appear to be similar across France, Italy and the UK^79^ (Appendix Table A3). However, coverage is reportedly unevenly distributed because screening is not routine and relies on the physician’s judgement.

In 2019, universal HBV childhood vaccination, as advocated by WHO in 2014, had been implemented at national level in 49 of the 53 Member States of the WHO European Region. In the four others (Denmark, Finland, Iceland and Sweden), vaccination covered only high-risk groups.^80^ In Italy and the UK (the only two countries with refugee and migrant-specific guidelines), the guidelines did not include catch-up vaccination for those born before the start of universal infant immunization in their country of origin contrary to WHO recommendations.^47–49^ Adult HBV vaccination programmes in these two countries target refugees and migrants in high-risk groups only (Appendix Table A3).

A 2014 survey investigating awareness in healthcare professionals of HBV and HCV screening and management in Germany, Hungary, Italy, the Netherlands, Spain and the UK found that, only in the Netherlands were asylum seekers from high-prevalence areas commonly HBV vaccinated, and only in Spain were refugees and migrants were commonly vaccinated^81^ (there is no vaccine available for HCV). A high proportion of respondents from all six countries lacked awareness of current vaccination practices for refugees and migrants from high HBV prevalence areas. Surprisingly, this included respondents from Italy and the UK, where specific recommendations are available.^47^^,^^48^^,^^82^

### Implementation of TB, HIV, HBV and HCV infection services for refugees and migrants: barriers and facilitators

Even where free access to healthcare is available to refugees and migrants, our review identified systemic barriers to access (see country examples, Appendix Table A4). Table 3 presents the results of a thematic analysis of key barriers and facilitators operating at macro- (policy or transnational), meso- (community or service) and micro-levels (structural, sociocultural or socioeconomic)^83^ identified by our search.

At the macro-level, the need for political commitment to the formation and implementation of national policy was frequently emphasized. Many documents highlighted the importance of strengthening approaches to data collection on fefugee/migrant status in health systems across Europe to provide a Regional evidence base on TB/HIV/HBV/HCV infection in refugees and migrants for monitoring and evaluation within national health systems.^3^^,^^84^^,^^85^ WHO has highlighted an urgent need to integrate the migration health data into every national health information system (HIS) to make data available for policy planning and for implementing refugee- and migrant-sensitive policies and intervention programmes.^86^ This would increase the migration health data availability and would support data comparison with the host population. The integration of a set of core variables into HIS (including country of birth, country of citizenship, year and month of arrival and country of birth of both parents) will facilitate the disaggregation of HIS data by migratory status.

Other barriers to data collection include difficulties in accessing key refugee and migrant subgroups, mistrust and language barriers, the large and varied choice of health indicators and definitions of health and migrant. There is also limited data sharing between agencies, partly because of incompatible software systems and data protection regulations at both national and regional levels. Data protection and separation between the health and legal systems are imperative to promote confidence in migrants of these data collection systems.^86^

At the meso-level, stigma associated with TB/HIV/HBV/HCV infection diagnosis was frequently highlighted as a barrier for refugees and migrants. Articles discussed the importance of involving and engaging affected communities, peer support and community champions and the importance of clear health information messaging. They emphasized that access to services should be promoted by removing financial barriers, such as upfront payments, and that service use should be facilitated by offering culturally relevant care for multiple health conditions, offering the flexibility to meet the target populations’ needs.

At the micro-level, sociocultural factors included removing language barriers and providing appropriate counselling and education. Refugees and migrants may experience fear and lack of trust, particularly about their right to remain in the host country, and may have limited knowledge of the local health and social care services, which prevents them from identifying the appropriate service for their health condition. Documents recommended that refugees and migrants should be involved in healthcare decision-making and delivery as well as in developing health education to improve health literacy on the prevention, treatment and care for TB/HIV/HBV/HCV infection. This would increase refugees' and migrants’ knowledge, awareness and, subsequently, use of health services as well as awareness of their rights and entitlements.

On 4 March 2022, the EU agreed to activate the Temporary Protection Directive, offering immediate, temporary protection for displaced people from outside the EU, which was intended to be used in exceptional circumstances when the regular EU asylum system struggles to handle a ‘mass influx’ of refugees. This protection for people fleeing the war in Ukraine includes a temporary residence permit and access to healthcare services.^21^ However, implementation of this cannot be guaranteed, with reception countries having varied levels of preparedness and capacity within their health systems and burden of infectious diseases, including TB/HIV/HBV/HVC remaining high in Ukraine.^87^ Reception countries must offer vaccination and screening services as well as ensure continuity of care for those currently under treatment, but the scale up influx (>6 600 000 refugees from Ukraine recorded across Europe, >3 800 000 of these registered for Temporary Protection as of 17 August 2022^88^) requires more support from other countries.^87^

### Cost-effectiveness of targeted interventions for refugees and migrants

Most identified cost-effectiveness analyses of targeted interventions for refugees and migrants evaluated TB interventions; substantial research gaps exist for HIV/HBV/HCV infection (Appendix Table A5), which must be addressed as such analyses can provide explicit evidence to motivate decision-makers to change current policies.

Even for TB, many sources stated that considerable knowledge gaps remain; further analyses and empirical research to inform reliable inputs for TB cost-effectiveness models are required. Knowledge gaps relate to disaggregating cost-effectiveness by refugee/migrant type, reason for migration and factors such as age and migration trajectory, which screening method to use and which point (country of origin, transit country or country of settlement) and location are best for screening.^89^ While there has been increasing focus on pre-entry TB screening programmes in low-incidence host countries because it is perceived to be a more cost-effective approach, as mentioned above, it only targets a subset of refugees and migrants.

Evidence is still lacking on the most cost-effective or efficient approaches for TB detection and the continuum of care across national borders. Reviewed documents recommended addressing these gaps by obtaining prospective, multicentre data on LTBI prevalence in refugees and migrants and assessing the performance of screening tools and the outcomes of screening in different locations.^89^

The most cost-effective approach is likely to involve a combination of interventions: targeted pre-arrival screening for active TB followed by post-arrival screening for LTBI in refugees and migrants from settings with an intermediate–high TB burden.^90^ However, evidence is limited on the cost-effectiveness of different screening approaches. Therefore, further exploration of these combinations of interventions is warranted.

There remains a lack of health economic evaluation of refugee- and migrant-specific HIV interventions such as dedicated screening and PrEP programmes. There is growing evidence of the cost-effectiveness of HBV screening for higher-risk refugees and migrants, although the determination of the screening threshold requires more evidence. Less evidence is available for refugee- and migrant-specific HCV interventions. Combined HBV/HCV may increase the cost-effectiveness and yield of prevention efforts but requires further research.^32^ Broadening focus to the evaluation of interventions for all four infections (TB/HIV/HBV/HCV) is a further important gap. The feasibility of an even wider scope to consider a more comprehensive health screening, including other infections (e.g. parasitic worms) and non-communicable conditions such as diabetes and cardiovascular disease should, be explored. However, the cost-effectiveness of increasing the number of conditions depends on the costs of testing (including additional consultation time), the prevalence of each condition among those tested and its health- and social care-related consequences. In practice, the consultation time per patient may limit the range of conditions that could be considered at one visit.

## Discussion

Despite the WHO and UN General Assembly calls for the provision of targeted, non-discriminatory health protection services for refugees and migrants in both transit and destination countries,^17^^,^^91^ our review identified relatively few (*n* = 15) primary policy documents pertaining to TB/HIV/HBV/HCV infection and refugees and migrants from the WHO European Region countries. Data were particularly lacking from some Member States, in particular, those in eastern Europe and central Asia. Many of the included documents captured by our search were surveys of relevant healthcare professionals to ascertain the current national policies and guidance regarding refugees and migrants; however, these covered only a narrow range of European countries. Even where specific national guidelines were available, such as for Italy and the UK, the studies typically revealed a lack of awareness of these guidelines by relevant healthcare and public health professionals.

It was difficult to gauge whether formal national policies for refugees and migrants and TB/HIV/HBV/HCV infection exist for many countries or were just inaccessible (e.g. non-English and non-Russian publications only, no online access). Future searches of the WHO European Region ministry of health websites with no language restrictions, combined with expert interviews in key receiving countries, are needed to identify other potentially relevant documents to enable a fully comprehensive review of all relevant policy documents.

From the evidence on national policies gathered, we found significant heterogeneity in the implementation of TB/HIV/HBV/HCV infection prevention, diagnosis, treatment and care measures across the WHO European Region. Furthermore, official national policies and guidelines often do not align with WHO recommendations and are not easily accessible, and considerable knowledge gaps remain, which are needed to provide the evidence base on which to build interventions and policies.

We have summarized our findings with lists of key policy considerations (Box 1) and directions for future research (Box 2). Screening and treatment for TB/HIV/HBV/HCV infection must be incorporated into refugee and migrant screening programmes (e.g. at the first point of contact for newly arrived refugees and migrants with health services in the host country). This should be done in an accessible and culturally sensitive manner as part of a basic, free package of care. Improved cross-border collaboration for infection screening and care is needed along the entire migration trajectory, with a focus on implementing a minimum package of screening and care.^13^ Many publications advocated developing a more holistic approach to refugee and migrant health across the Region which recognizes refugees’ and migrants’ right to health and aims to remove the legal, social and cultural barriers to health services to improve the control of TB/HIV/HBV/HCV infection. This would require a multisectoral approach, with support at all levels of government.

More transparent and accessible reporting of national policies and guidelines on the prevention, diagnosis, treatment and care of TB/HIV/HBV/HCV infection in refugees and migrants is required for all Member States in the Region, with provision of the evidence base upon which these policy decisions are based. Finally, political engagement is essential to drive changes in the national legislation to ensure an equitable and universal access to the diagnosis and care for infectious diseases and to reduce the social risk factors for both refugees and migrants and host populations. Therefore, dialogues should be fostered with Member States whose policies do not align with WHO and ECDC recommendations for delivering TB/HIV/HBV/HCV infection services to refugees and migrants to understand the reasons for current policies and to identify the macro-level barriers to policy change.

**Box 1. Policy considerations**

- Support the WHO in initiating discussions with WHO European Region Member States whose policies do not align with WHO and ECDC recommendations on delivering TB, HIV, HBV and HCV infection services to refugees and migrants to facilitate policy change;
- Improve the online accessibility of national guidelines and policies on TB, HIV, HBV and HCV infection prevention, diagnosis, treatment and care of refugees and migrants, including the evidence base informing policy development;
- Develop and implement schemes to improve awareness among refugees and migrants of relevant policies and guidelines that promote patients' rights;
- Support national initiatives to address misinformation, stigma and discrimination regarding refugees and migrants and encourage and improve inclusive approaches, such as promoting health literacy;
- Conduct comprehensive assessments of barriers to health (including language, cultural and physical barriers, legal barriers and entitlements, fear of registration and deportation, out-of-pocket payments, discrimination and stigma, insufficient training for health and social services providers) with the involvement of refugee and migrant groups.
- Strengthen routine health data collection to improve monitoring of migration health data and optimise targeted screening strategies through:

– integrating migration health data into national health information systems;
introducing dynamic reporting of estimates of infectious disease prevalence in different refugee and migrant populations;
disaggregating health data by refugee and migrant subgroups using WHO-recommended core variables (country of birth, country of citizenship, month and year of arrival, and country of birth of both parents) plus a second set of recommended variables to enable disaggregation by subgroups of refugees and migrants (reasons for migration, knowledge of official language(s) of host country, ever-resided abroad and legal status);

- Strengthen health systems by:

providing awareness training on refugee and migrant health for healthcare practitioners to increase their adherence to national policies and guidelines; and
developing initiatives to improve service delivery for refugees and migrants by removing barriers to access and utilizing facilitators; and

- Increased and longer-term support for reception countries hosting large numbers of refugees from Ukraine to ensure healthcare access

Box adapted from Box 1, WHO Health Evidence Network synthesis report 74.^24^

**Box 2. Future research**

To provide an adequate evidence base for WHO European Region Member States to design and implement effective policies and guidelines, future research efforts should include:

- analysing patterns of TB, HIV, HBV and HCV infection prevalence and transmission and defining high-risk thresholds for these diseases among refugee and migrant populations;
- qualitative studies of refugees' and migrants’ perceptions of available services to assess the impact of national policy and guidance documents on refugees' and migrants’ rights, access to care and treatment adherence;
- conducting well-parameterised cost-effectiveness analyses to plan efficient targeted screening approaches; and
- performing regular surveys at regional level to catalogue national policies and guidelines and their adherence to WHO/ECDC recommendations.

Box adapted from Box 2, WHO Health Evidence Network synthesis report 74.^24^

## Supplementary Material

Baggaley_et_al_Appendix_taac136
