# Supplementary material for: National policies for delivering tuberculosis, HIV and hepatitis B and C virus infection services for refugees and migrants among Member States of the WHO European Region
Source: J Travel Med. 2022 Nov 25;30(1):taac136. doi: 10.1093/jtm/taac136 (PMC9940698; doi:10.1093/jtm/taac136)
Supplement: Baggaley_et_al_Appendix_taac136 [file baggaley_et_al_appendix_taac136.docx]

**Appendix, Baggaley *et al***

**Systematic review methods**

**Databases and websites**

Searches of peer-reviewed literature in four English-language databases (Embase, Health Management Information Consortium, Medline and OpenGrey) from inception to present were carried out on 30 November 2020. In addition, a targeted search for relevant published and unpublished documents in Russian in the Electronic Library of Scientific Publications (e-library.ru; integrated into the Russian Science Index) was conducted using the defined search terms in Russian on March 23^rd^, 2021. Grey literature was also obtained by searching the websites of ECDC, WHO and ministries of health websites of WHO European Region Member States. Additional relevant articles were identified by contacting members of WHO team networks for TB (*n* = 373), hepatitis and HIV (*n* = 633), and Migration (*n* = 44).

**Search terms**

Search terms were related to TB, HIV, HBV, HCV, migration, and policy or guidance documents (in English or Russian).

The following is a representative search strategy (Medline):

human migration/ or migration.mp. or migrant*.mp. or "transients and migrants"/ or "emigrants and immigrants"/ or immigra*.mp. or expatriate*.mp. or refugees/ or refugee*.mp. or departee.mp. or "emigration and immigration"/ or emigr*.mp. or asylum.mp. or foreign-born.mp. or foreign born.mp. or foreign worker*.mp. or international student*.mp. or human traffick*.mp. or Human Trafficking/ or people traffick*.mp. or sex traffick*.mp. or ((wom?n or child* or men or man) adj2 traffick*).mp. (tuberculosis or tb or (LTBI or latent tuberculosis) or (MDRTB or XDRTB or XDR-TB)).mp. or exp Tuberculosis, Multidrug-Resistant/ or exp Tuberculosis exp HIV/ or (HIV or ("human immun*" and virus)).mp. or (("acquired immun*" and syndrom*) or "aids virus" or "HIV/AIDS").mp. or Acquired Immunodeficiency Syndrome/ or PLWH*.mp. or antiretroviral*.mp. or Anti-Retroviral Agents (HCV or HBV or HBsAg or viral hepatiti*).mp. or exp Hepatitis, Viral, Human/ or (hepatiti* adj3 virus).mp (Europe/ and World Health Organization/) or WHO european region.mp. or (Albania or Andorra or Armenia or Austria or Azerbaijan or Belarus or Belgium or Bosnia or Herzegovina or Bulgaria or Croatia or Cyprus or Czechia or Denmark or Estonia or Finland or France or Georgia or Germany or Greece or Hungary or Iceland or Ireland or Israel or Italy or Kazakhstan or Kyrgyzstan or Latvia or Lithuania or Luxembourg or Malta or Monaco or Montenegro or Netherlands or Norway or Poland or Portugal or Moldova or Romania or the Russian Federation or Russian Federation or San Marino or Serbia or Slovakia or Slovenia or Spain or Sweden or Switzerland or Tajikistan or Macedonia or Yugoslavia or Yugoslav or Türkiye^[[1]](#footnote-1)^ or Turkmenistan or Ukraine or United Kingdom or United Kingdom or England or Ireland or Scotland or Wales or Great Britain or Uzbekistan or EEA or EU or European Union or Europe or European Economic Area).mp. exp clinical pathway/ or exp clinical protocol/ or exp consensus/ or exp consensus development conference/ or exp consensus development conferences as topic/ or critical pathways/ or guidelines as topic/ or exp practice guideline/ or practice guidelines as topic/ or health planning guidelines/ or (guideline or practice guideline or consensus development conference or consensus development conference, NIH).pt. or ((practice or treatment* or clinical) adj guideline*).ab. or (CPG or CPGs).ti. or consensus*.ab. /freq=2 or polic*.mp. or (Health Policy/ or Organizational Policy/ or Policy/ or Policy-making/ or Public Policy/) or technical report*.mp. or technical report.pt. [mp=ti, ab, hw, tn, ot, dm, mf, dv, kw, fx, dq, nm, kf, ox, px, rx, ui, sy]

**Study selection**

Studies were selected according to PRISMA guidelines^1^ (Figure A1). Titles and abstracts of all citations identified through the searches were screened by one reviewer (JN or RFB); duplicate screening was performed for a subset of documents, with discrepancies resolved by consensus. The full texts of relevant publications were screened to identify papers for inclusion.

Inclusion criteria were:

- reviews, viewpoints/editorials, policy documents, guidelines and grey literature (including national/international reports and case studies) reporting on policies or guidelines relevant to the prevention, diagnosis, treatment or care of TB, HIV/AIDS, or hepatitis B or C among migrants^[[2]](#footnote-2)^ in the WHO European Region;
- primary research on the implementation or effectiveness of policies and guidelines;
- published after 2010 (for Member States with few identified documents, older documents were also included); and
- published in English or Russian.

Exclusion criteria were:

- does not mention refugees, migrants or asylum seekers in the main text;
- about countries outside the WHO European Region;
- report the results of primary research, except for primary research collating information on policies or study participants' opinions on policies; or
- university thesis.

**Data extraction and assimilation**

Articles were organised and deduplicated using Rayyan (an app designed for systematic reviews). A data extraction form was developed based on the study aims to extract data in the following categories: doi, year of publication, authors, title and abstract, language, organization, type of document, methods, disease, focus (prevention, diagnosis, treatment or care), migrant population, country, and relevant policy content (legislation, policy, or guideline; recommendations; alignment with WHO-recommended actions; barriers and facilitators to implementation of guidance; cost-effectiveness or national funding allocation; and gaps). The form was piloted by two reviewers prior to full data extraction. For validation of data extraction, a subset of extracted documents was reviewed by each reviewer. Framework analysis was conducted to identify key themes across policy content categories.

**Fig. A.1** Flowchart of included studies: summary of literature search and screening process

Relevant articles identified through consultation with WHO co-authors and collaborators and through review of included articles (*n* = 63)

Records after duplicates removed

(*n* = 1390)

Records excluded

(*n* = 912)

Full-text articles excluded, with reasons

(*n* = 273)

Full-text articles assessed for eligibility

(*n* = 478)

Studies included in qualitative synthesis

(*n* = 268)

Titles and Abstracts screened

(*n* = 1390)

Records identified through database searches in English and Russian:

Medline & Embase (*n* = 977)

HMIC (*n* = 28)

OpenGrey (*n* = 66)

ECDC (*n* = 97)

## Screening

## Eligibility

## Identification

Additional records identified from other sources:

Google searches of Ministry of Health websites (*n* = 30)

WHO email to TB/HIV/HEP/Migrant networks (*n* = 53)

Records identified through searching the Russian literature:

e-library.ru (*n* = 142)

HMIC: Health Management Information Consortium.

**Table A1.** Summary of identified national screening policies for active and latent TB infection among migrants for the WHO European Region.

| **Country** | **Screening strategy** | **Population screened** | **Age range (years)** | **Active TB screening** | |  | **Latent TB screening** | |
| --- | --- | --- | --- | --- | --- | --- | --- | --- |
|  |  |  |  | **TB incidence in country of origin: threshold for screening^a^** | **Screening method** |  | **TB incidence in country of origin: threshold for screening^a^** | **Screening method** |
| Belgium^2^ | NA | Asylum seekers | <5/pregnant | All | CXR for TST positives |  | All | TST |
|  |  |  | ≥5 (exc pregnant women) | All | CXR |  | – | None |
|  |  | Other migrants | – | – | None |  | – | None |
| Finland^2^ | NA | Asylum seekers | All | >50 or from conflict areas | CXR |  | Partial^b^ | TST |
|  |  |  | All | <50 | CXR |  | – | None |
|  |  | Other migrants | All | >50 | CXR |  | – | None |
| France^3-5^ | Post-arrival medical centre | Long-stay migrants (>3 months)^c^ | All | >40 | CXR |  | “High”^d^ | NA |
| Germany^2,6^ | Post-arrival | Asylum seekers in community/reception centres | >15/ pregnant | All | TST/IGRA followed by further diagnostics |  | – | None |
|  |  |  | ≥15 (exc pregnant women) | All | CXR |  | – | None |
| Greece^2^ | NA | Asylum seekers/ undocumented migrants in reception centres | All | All | Interview |  | – | None |
|  |  | Asylum seekers entering hosting structures | All | All | CXR |  | All | TST |
|  |  | Other migrants | All | All | CXR |  | All | TST |
| Ireland^4,7^ | Post-arrival | All migrants | <16/ pregnant | ≥40 | TST followed by CXR + sputum |  | >40 | TST |
|  |  |  | ≥16 | 40-500 | CXR |  | – | None |
|  |  |  | 16–35 | >500^e^ | CXR + TST |  | >500 | TST |
| Italy^2,4,8^ | Post-arrival, secondary reception centre/health centre | All migrants | – | – | Partial^f^ |  | >100^g^ | TST/IGRA |
| The Netherlands^2^ | Post-arrival central reception centres | Asylum seekers | <18 | >50 | CXR |  | TBC^h^ | TBC^g^ |
|  |  |  | ≥18 | >50 | CXR |  | – | None |
|  | Post-arrival, public health service | Other migrants | <18 | >50 | CXR for those with LTBI |  | All | IGRA |
|  |  |  | ≥18 | >50 | CXR^i^ |  | – | None |
| Norway^9,j^ | Post-arrival | Asylum seekers | <15 | All | CXR for those with LTBI |  | All | IGRA |
|  |  |  | 15-34 | All | CXR |  | >200^k^ | IGRA |
|  |  |  | ≥35 | All | CXR |  | – ^k^ | None |
|  |  | Other migrants | <15 | >40 | CXR for those with LTBI |  | >40 | IGRA |
|  |  |  | 15-34 | >40 | CXR |  | >200 ^k^ | IGRA |
|  |  |  | ≥35 | >40 | CXR |  | – ^k^ | None |
| Russian Federation^10^ | Post-arrival medical centre, primary and secondary care centres | Labour migrants, migrants receiving care in primary or secondary healthcare systems | All | NA | CXR  (pregnant women: sputum microscopy) |  | NA | NA |
|  |  |  |  |  |  |  |  |  |
| Spain^2^ | NA | All migrants <5 years since arrival | All | “High”^l^ | CXR for those with LTBI |  | “High” ^l^ | TST/IGRA |
| Sweden^2^ | Post-arrival, primary care centre | Asylum seekers | All | >100 | CXR for those with LTBI^m^ |  | >100^j^ | TST/IGRA^m^ |
|  | NA | Other migrants | – | – | None |  | – | None |
| Switzerland^2^ | NA | Asylum seekers | All | All | Interview |  | – | None |
|  |  | Other migrants | – | – | None |  | – | None |
| United Kingdom^2,11-14^ | Pre-entry^n^ | Long-stay migrants (>6 months) | <11 | >40 | Interview |  | >40 | TST/IGRA |
|  |  |  | 11–15 | >40 | CXR |  | >40 | TST/IGRA |
|  |  |  | 16–35 | >40 | CXR |  | >150^m^ | IGRA |
|  |  |  | >35 | >40 | CXR |  | – | None |

Table adapted from Tables 3 and 4, WHO Health Evidence Network synthesis report 74.^15^

ATT – antitubercular treatment; CXR – chest X-ray; EU – European Union; exc – excluding; IGRA – interferon gamma release assay; LTBI – latent TB infection; NA – not available; TBC – to be confirmed; TST – tuberculin skin test.

^a^ Cases per 100,000 population; WHO estimated threshold.

^b^ Latent TB infection testing of children <7 years, if TST-negative with no BCG scar.

^c^ To be screened within 4 months of arrival. Active and latent TB infection screening strategy in the process of being at the time of writing the review used as our source (ECDC guidance, 2018^4^).

^d^ For children <15 years only.

^e^ 16-35 years plus either from country of origin >500/100,000 TB incidence or sub Saharan Africa.

^f^ Kunst et al., 2017^2^ report that no national policy for active TB screening of migrants exists in Italy, but triaging on symptoms followed by CXR or sputum examination is performed in several centres in the country. ECDC 2018^4^ report that guidelines for Italy are that all migrants from high incidence (>100/100,000 population) countries be offered TST; TST/IGRA-positive subjects should be offered CXR and other diagnostic tests and treatment provided appropriately (for active TB or preventive treatment).

^g^ For migrants expecting to stay for ≥6 months.

^h^ For the Netherlands, latent TB infection screening strategy for children (<18 years) was yet to be confirmed at the time of writing the review used as our source (Kunst et al., 2017^2^). TB incidence threshold on country of origin for screening set as >50/100,000 population but yet to be implemented.^2^

^i^ The Netherlands offers half-yearly follow-up CXR screening for 2 years for migrants from high incidence countries.^2^

^j^ Information provided by scientific literature^9^ reporting official policy: Norwegian Institute of Public Health. Guidelines for the prevention and control of tuberculosis; Chapter 4.2.1. https://www.fhi.no/nettpub/ tuberkuloseveilederen/. Accessed 29 Jan 2019 [in Norwegian].

^k^ The stated TB incidence threshold plus Eritrea and Afghanistan countries of origin. In addition, LTBI testing is recommended in Norway to all migrants with medical risk factors for progression to TB disease.

^l^ In Catalonia, a high-incidence country is defined as any country with more than three times the local TB incidence.

^m^ LTBI testing in asylum seekers aged 735 years in Sweden is performed to identify those eligible for CXR screening, not for preventive treatment.

^n^ In the United Kingdom of Great Britain and Northern Ireland, screening for active TB previously took place at airports; however, in 2012 this system was replaced with pre-entry screening for applicants who apply for a United Kingdom visa for >6 months and are resident in a country with an incidence of >40 cases/100,000 population. Pre-entry screening is for active TB only and applies to individuals whose country of residence in the previous 6 months has been higher than the incidence threshold. Active TB screening for pregnant women is a symptom screen, contact history and physical examination if required, with CXR in the second or third trimester if the applicant consents. For further details of screening for pregnant women and children <11 years, see the United Kingdom technical instructions^12^.

^m^ 16-35 years plus either born or spent >6 months in a high TB incidence country (>150/100,000) or any country in sub Saharan Africa. Individuals also must have no history of TB (treated or untreated) and have never been tested for LTBI in the United Kingdom.

*Sources*: includes information from Bozorgmehr et al., 2017^6^; Kunst et al., 2017^2^; European Centre for Disease Prevention and Control, 2018^4^; Kehr et al., 2012^3^; and Garner-Purkis et al., 2019.^16^

**Table A2.** Summary of national policies within the WHO European Region regarding HIV testing and care relating to migrants identified by the search.

| **Country** | **Testing** | **Discrimination/stigmatisation** |
| --- | --- | --- |
| United Kingdom | Specific guidance for HIV screening and management for migrants^17^  Routine (not annual) testing for the following migrant types:   - migrants newly registering with a GP in an area with HIV prevalence >0·2% - migrants from countries with HIV prevalence >1% - all men and women reporting sexual contact abroad or in the Untied Kingdom with individuals from countries of high HIV prevalence   Additional guidance is given for persons born in a high-prevalence country: regular HIV, and sexually transmitted infection (STI) screening is advised if the individual is having unprotected sex with new or casual partners.  Black African testing rates published^18^ | Recommendations for HIV testing of migrants^19^:   - genuinely voluntary and free of any coercion; testing programmes must respect international guidelines on confidentiality, counselling and consent. - should not be required of workers, including migrant workers; test results should be confidential and not endanger access to jobs, tenure, job security or opportunities for advancement. - Workers, including migrant workers, should not be required by countries of origin, transit or destination to disclose HIV-related information about themselves or others; access to such information should be governed by existing rules of confidentiality |
| France | Migrants recommended to be screened annually if they have a sexual partner from a HIV endemic region^20^  Migrant testing rates published^18^ |  |
| Greece | Migrant testing rates published including for undocumented migrants^18^ |  |
| Luxembourg | Asylum seeker testing rates published^18^ |  |
| Cyprus | No specific HIV programmes targeted at migrants identified.^21^ HIV screening of all new arrivals takes place in the immigration centre in Menogia. Although, testing is voluntary, new arrivals are only permitted into the immigration centre if they consent to testing. |  |
| Russian Federation, Turkmenistan |  | - HIV testing is required for work permits - HIV testing is required for study permits - HIV testing or disclosure is required for certain permits or entry less than 90 days - HIV testing is required for residency permits (for stays longer than 90 days) - entry and stay for less than 90 days are prohibited on the basis of HIV status - residency permits are denied (for stays longer than 90 days) on the basis of HIV status - non-national PLWH are deported on the basis of HIV status^22^ |
| Bulgaria, Czechia |  | Travel restrictions recently removed^23-25^ |
| Bosnia and Herzegovina, Kyrgyzstan, Ukraine |  | Prohibit short- and/or long-term stay on the basis of HIV status^22^ |
| Azerbaijan, Israel, Kazakhstan |  | Require HIV testing or disclosure for certain types of entry, study, work and/or residency permits^22^ |

GP – general practitioner; HBV – hepatitis B virus infection; HCV – hepatitis C virus infection; PrEP – pre-exposure prophylaxis; STI – sexually transmitted infections; TB – tuberculosis.

**Table A3.** Summary of national policies within the WHO European Region regarding HBV and HCV testing and care relating to migrants identified by the search.

| **Country** | **HBV testing** | **HBV vaccination** | **HCV interventions** |
| --- | --- | --- | --- |
| United Kingdom | Screening recommended for migrants from countries with intermediate/high prevalence of chronic HBV infection (HBsAg prevalence >2%).^26^  For infants born to HbsAg-positive mothers: recommended routine vaccination with appropriate follow-up.^26a^ | Universal HBV childhood vaccination does not include catch-up vaccination for those born before the start of universal infant immunization in their country of origin, contrary to WHO recommendations^26^  HBV adult vaccination programme target migrants in high-risk groups: those travelling for extended periods to HBV-endemic countries, including those who have received medical treatment abroad^26^ | Screening for chronic HCV infection should be considered for migrants from countries with a higher prevalence of HCV^27^  Country-specific guidance on exposure avoidance to prevent HCV infection^27^  Screening recommended for pregnant mothers regardless of country of origin^c^ |
| Italy | Screening advised for migrants from high prevalence areas^28a,b^ | Universal HBV childhood vaccination does not include catch-up vaccination for those born before the start of universal infant immunization in their country of origin, contrary to WHO recommendations^29^  HBV adult vaccination programme target migrants in high-risk groups: those originating from highly endemic areas, drug users, non-immunised prison inmates, partners of infected individuals, and patients with non-HBV-related chronic liver disease^29^ | Screening for chronic HCV infection recommended for migrants from areas of high prevalence e.g., Egypt, the Middle East, Pakistan^29d^  Screening recommended for pregnant mothers regardless of country of origin^c^ |
| France | Newly arrived irregular migrants, asylum seekers and residence permit applicants undergo a medical examination but this does not include testing for chronic HBV infection.^28^ However, increased testing in France was achieved following a national action plan for 2009–2012^28a^ |  | Screening for chronic HCV infection has become more widespread since 2009, when the health budget for migrants included testing by state healthcare centres for HCV but not HBV^28^  Screening recommended for pregnant mothers regardless of country of origin^c^ |

^a^ A position paper by Santilli^28^ reported that evidence suggests that screening policies for HBV infection are similar for France, Italy, and the United Kingdom.

^b^ Threshold for high HBV prevalence not stated by Santilli.^28^

^c^ Aligns with the WHO goal of 90% HBV screening coverage for pregnant women.^30^

^d^ However, HCV threshold for screening not indicated.

**Table A4.** Systemic barriers to migrant access to healthcare – examples from WHO European Region countries.

| **Country** | **Barrier description** |
| --- | --- |
| United Kingdom | Anyone HCV-positive can receive treatment, but guidance does not specify whether this is free for all migrant types (i.e. undocumented as well as documented)^27^ |
| Italy | Free access to the national healthcare system (emergency and preventive services) is guaranteed, but free access to additional services depends on type of residence permit^28^: migrants with chronic diseases have free access to healthcare, whereas those who have been granted refugee status have to pay to access healthcare services (similar to Italian citizens). |
| France | Irregular migrants have free access to healthcare but under several conditions: they must prove residency in France for >3 months, have a place of residence and have submitted an application with the reception system (which requires the help of an expert).^28^ Waiting times to obtain health coverage can take 5–9 months, during which migrants cannot access free treatment, which is often provided by humanitarian associations. |
| Russian Federation | Access to healthcare depends on migrant status: (i) labour migrants with temporary or permanent resident permission plus non-labour migrants with permanent resident permission; and (ii) seasonal labour migrants and non-labour migrants with temporary resident permission. State-funded healthcare coverage is accessible for labour migrants from Eurasian Economic Union in the Russian Federation provided they have registration in the place they live and an official contract with an employer in the Russian Federation. |

**Table A5.** Summary of cost-effectiveness analyses for TB, HIV and HBV and HCV infection interventions for migrants identified by the review.

| **Tuberculosis** | **HIV** | **HBV/HCV** |
| --- | --- | --- |
| LTBI screening of migrants is cost-effective and becomes more so when targeted to migrants at the highest risk of infection, although currently there is no consensus on the optimal TB incidence in country of origin threshold for screening^31,32^ | A 2018 systematic review of the effectiveness and cost-effectiveness of HIV screening in migrants in the EU/EEA found no data on cost-effectiveness or resource requirements.^33^ | Until recently, evidence lacking on the cost-effectiveness of screening for viral hepatitis among migrants.^34,35^  Now growing evidence that HBV screening is cost-effective when targeted to migrants from high prevalence (generally ≥2%) countries^36^ and likely also intermediate prevalence countries.^37,38^ |
| Cost-effectiveness of active TB screening: evidence is less clear than for LTBI. Indiscriminate CXR screening of migrants is inefficient and not cost-effective^39-43^  Likely cost-effective only in specific contexts and for high-risk migrant populations. Prioritizing interventions and targeting screening to higher-risk groups can improve cost-effectiveness and is endorsed by the WHO^44^ | 2021 costing analysis: ART provision for migrants to Russian Federation^45^: it may be three times cheaper to provide ART to HIV-positive international migrants for one year than to pay for a three-week hospital stay caused by an AIDS-related opportunistic infection: a likely consequence of barriers to ART uptake for migrants. | Less evidence is available on cost-effectiveness of interventions targeted to migrants for chronic HCV infection^36^; however assessment of HCV testing for migrants in the United Kingdom was reported to be cost-effective.^46^ |

**Table A6.** Summary of cost-effectiveness analyses of diagnostic tests for LTBI screening in migrant groups

| **Diagnostic test evaluated** | **Source** | **Finding** | **Level of evidence^a^** |
| --- | --- | --- | --- |
| TST | Two cost-effectiveness studies reporting on recently arrived migrants from high TB burden countries^47^ | TST (≥ 10 mm) and subsequent treatment for newly arrived adult migrants is highly cost-effective for LTBI diagnosis (compared with no screening)^b^ | Weak |
|  | Two cost-effectiveness studies reporting on recently arrived migrants^48^ | TST (≥ 5 mm) for migrants is cost-effective for diagnosis of LTBI (compared with TST (≥ 5 mm) positive, followed by IGRA)^c^ | Weak |
| IGRA | Five cost-effectiveness studies reporting on recently arrived migrants from high TB burden countries^39^ | IGRA screening of adult migrants is moderately cost-effective for LTBI diagnosis (compared with no screening)^b^ | Weak |
|  | Eight cost-effectiveness studies reporting on selected risk groups^49^ | IGRA screening of high-risk groups (e.g. healthcare workers, migrants from high-incidence countries and close contacts) is moderately cost-effective^b^ | Weak |
| TST and IGRA | Eight cost-effectiveness studies^49^ | IGRA screening of TST-positive individuals in high-risk groups (e.g. healthcare workers, migrants from high-incidence countries and close contacts) is cost-effective^d^ | Weak |
|  | Cost-effectiveness analyses (number included not stated)^50^ | From a healthcare perspective, regardless of the population group at risk, LTBI screening is most cost-effective when done using TST, with a positive TST followed by IGRA.  Cost-effectiveness is comparable for IGRA alone or TST alone.  From a societal perspective, using only IGRA is often the most cost-effective option, because it requires only one visit for testing | NA |

NA: not applicable.

^a^ As assessed by ECDC.^51^

^b^ Cost-effectiveness was defined using an incremental cost-effectiveness ratio: <$20,000, highly cost-effective; $20.000–$100.000, moderately cost-effective; > $100,000, not cost-effective.

^c^ An incremental cost-effectiveness ratio of <£20,000 was considered cost-effective. The review included primary studies conducted in low- and high-incidence settings.

^d^ Primary studies used different willingness-to-pay thresholds to identify cost-effective interventions.

*Source*: adapted from European Centre for Disease Prevention and Control, 2018.^51^

| Box A1. Action Plan for the Health Sector response to HIV in the WHO European Region: regional targets for HIV management  Targets for prevention are to:   - reduce new infections by 75% (or an appropriate numerical target for low-prevalence countries), including among key populations; - reduce mother-to-child transmission to less than 2% in non-breastfeeding populations and less than 5% in breastfeeding populations; and - reduce the rates of congenital syphilis and of child HIV cases due to mother-to-child transmission to 50 cases of less per 100,000 live births.   Targets for testing and treatment are for:   - 90% of people living with HIV to know their HIV status; - 90% of people diagnosed with HIV to be receiving ART; and - 90% of people living with HIV who are on ART to achieve viral load suppression.   Targets for AIDS-related deaths are to:   - reduce AIDS-related deaths to below 30,000 (contributing towards reducing global AIDS-related deaths to below 500,000); - reduce TB deaths among people living with HIV by 75% (or an appropriate numerical target for low-prevalence countries); and - reduce hepatitis B and C deaths among people co-infected with HIV by 10%.   The target for discrimination is for:   - zero HIV-related discriminatory policies and legislation.   The target for financial sustainability is to:   - increase the number of countries that are sustainably funded for the HIV response with increased domestic financing to more than 90%. |
| --- |

*Source*: WHO Regional Office for Europe, 2017.^30^

References

1. Moher D, Liberati A, Tetzlaff J, Altman DG, Group P. Preferred reporting items for systematic reviews and meta-analyses: the PRISMA statement. *PLoS Med* 2009; **6**(7): e1000097.

2. Kunst H, Burman M, Arnesen TM, et al. Tuberculosis and latent tuberculous infection screening of migrants in Europe: Comparative analysis of policies, surveillance systems and results. *International Journal of Tuberculosis and Lung Disease* 2017; **21**(8): 840-51.

3. Kehr J. Blind spots and adverse conditions of care: Screening migrants for tuberculosis in France and Germany. *Sociology of Health and Illness* 2012; **34**(2): 251-65.

4. European Centre for Disease Prevention and Control (ECDC). Public health guidance on screening and vaccination for infectious diseases in newly arrived migrants within the EU/EEA. Available from: <https://www.ecdc.europa.eu/en/publications-data/public-health-guidance-screening-and-vaccination-infectious-diseases-newly> Accessed 26th April 2022.; 2018.

5. La ministre des solidarités et de la santé. Direction générale de la santé. Instruction No. DGS/SP1/DGOS/SDR4/DSS/SD2/DGCS/2018/143 du 8 juin 2018 relative à la mise en place du parcours de santé des

migrants primo-arrivants. [Instructions relating to the implementation of health examinations for newly-arrived migrants]. Available at: <http://circulaires.legifrance.gouv.fr/pdf/2018/07/cir_43755.pdf> Accessed 13 October 2022. 2018.

6. Bozorgmehr K, Razum O, Saure D, Joggerst B, Szecsenyi J, Stock C. Yield of active screening for tuberculosis among asylum seekers in Germany: a systematic review and meta-analysis. *Euro surveillance : bulletin Europeen sur les maladies transmissibles = European communicable disease bulletin* 2017; **22**(12).

7. Health Protection Surveillance Centre, Ireland. Infectious Disease Assessment for Migrants. Dublin, Ireland. Published July 2015. Available at:

<https://www.hpsc.ie/a-z/specificpopulations/migrants/guidance/File,14742,en.pdf> Accessed 13 October 2022. 2015.

8. INMP, SIMM controlli alla frontiera. La frontiera dei controlli. Controlli sanitari all’arrivo e percorsi di tutela per i migranti ospiti nei centri di accoglienza. Sistema Nazionale per le Linee-guida. June 2017. Available at:

<http://www.inmp.it/lg/LG_Migranti-integrata.pdf> Accessed 13 October 2022. 2017.

9. Nordstoga I, Drage M, Steen TW, Winje BA. Wanting to or having to – a qualitative study of experiences and attitudes towards migrant screening for tuberculosis in Norway. *BMC Public Health* 2019; **19**(1): 796.

10. Нечаевой ОБ, Под ред. Рабочая группа высокого уровня по туберкулезу в Российской Федерации. Рекомендации по вопросам контроля за туберкулезом среди мигрантов [High-Level Working Group on Tuberculosis in the Russian Federation. Recommendations on tuberculosis control among migrants]. Москва: Издательство «Триада»; 2015 (in Russian).

11. Tuberculosis screening [website]. London: Public Health England; 2018 (<https://www.gov.uk/guidance/tuberculosis-screening>, accessed 15 September 2021). 2018.

12. PHE. Public Health England and UK Visas and Immigration. UK Tuberculosis Technical Instructions (UKTBTI) version 7. Available from: <https://assets.publishing.service.gov.uk/government/uploads/system/uploads/attachment_data/file/800099/UK_tuberculosis_technical_instructions_version_7.pdf> Accessed 13 October 2022. 2019.

13. PHE. Public Health England. Latent TB infection testing and treatment programme: Technical guidance and specification. Available from: <https://assets.publishing.service.gov.uk/government/uploads/system/uploads/attachment_data/file/807363/LTBI_technical_specification_and_guidance.pdf> Accessed 13th October 2022. 2019.

14. PHE. Public Health England and NHS England. Latent TB Testing and Treatment for Migrants: A practical guide for commissioners and practitioners. Available at: <https://assets.publishing.service.gov.uk/government/uploads/system/uploads/attachment_data/file/442192/030615_LTBI_testing_and_treatment_for_migrants_1.pdf> accessed 13th October 2022. 2015.

15. ECDC. European Centre for Disease Prevention and Control. Published 5th July 2022. Operational considerations for the provision of the HIV continuum of care for refugees from Ukraine in the EU/EEA. Available from: <https://www.ecdc.europa.eu/en/publications-data/operational-considerations-provision-hiv-continuum-care-refugees-ukraine-eueea> Accessed 22nd August 2022. 2022.

16. Garner-Purkis A, Hine P, Gamage A, Perera S, Gulliford MC, M.C. G. Tuberculosis screening for prospective migrants to high-income countries: systematic review of policies. *Public Health* 2019; **168**: 142-7.

17. HIV: migrant health guide - GOV.UK; 2020.

18. ECDC. European Centre for Disease Control and Prevention. HIV testing: monitoring implementation of the Dublin Declaration on partnership to fight HIV/AIDS in Europe and Central Asia: 2018 progress report. Available from: <https://www.ecdc.europa.eu/en/publications-data/hiv-testing-monitoring-implementation-dublin-declaration-partnership-fight> Accessed 25th March 2022. 2019.

19. International Labour Conference: presentation of the recommendation on HIV and AIDS - GOV.UK. 2020.

20. Austin T, Traversy GP, Ha S, Timmerman K. Canadian and international recommendations on the frequency of HIV screening and testing: A systematic review. *Canada communicable disease report = Releve des maladies transmissibles au Canada* 2016; **42**(8): 161-8.

21. Prevention ECfD, Control. Technical mission: HIV in Cyprus : 15–17 October 2014. LU: Publications Office; 2015.

22. UNDP Ua. Still Not Welcome. HIV-related travel restrictions. UNAIDS explainer. Available from: <https://www.unaids.org/sites/default/files/media_asset/hiv-related-travel-restrictions-explainer_en.pdf> Accessed 14th July 2021. 2019.

23. Assembly UNG, Committee T, Committee I-AS. UNAIDS. UNAIDS Report on the Global AIDS Epidemic 2010: Geneva: UNAIDS; 2010.

24. Lazarus JV, Curth N, Weait M, Matic S. HIV-related restrictions on entry, residence and stay in the WHO European Region: a survey. *Journal of the International AIDS Society* 2010; **13**(101478566): 2.

25. WHO. Scaling up HIV testing and counselling in the WHO European Region. Copenhagen: WHO Regional Office for Europe; 2010 (<https://www.euro.who.int/en/health-topics/communicable-diseases/hivaids/publications/2010/scaling-up-hiv-testing-and-counselling-in-the-who-european-region.-policy-framework>, accessed 15 September 2021). 2010.

26. Hepatitis B: migrant health guide - GOV.UK; 2020.

27. Hepatitis C: migrant health guide - GOV.UK; 2020.

28. Santilli C. Medical Care, Screening and Regularization of Sub-Saharan Irregular Migrants Affected by Hepatitis B in France and Italy. *Journal of immigrant and minority health* 2018; **20**(3): 668-73.

29. Almasio PL, Babudieri S, Barbarini G, et al. Recommendations for the prevention, diagnosis, and treatment of chronic hepatitis b and c in special population groups (migrants, intravenous drug users and prison inmates). *Digestive and Liver Disease* 2011; **43**(8): 589-95.

30. Action plan for the health sector response to viral hepatitis in the WHO European Region: World Health Organisation; 2017.

31. Lalvani A, Pareek M. Immigrant screening for TB: A missed opportunity to improve TB control in the United Kingdom. *Pathogens and Global Health* 2012; **106**(1): 5-7.

32. Pareek M, Watson JP, Ormerod LP, et al. Screening of immigrants in the UK for imported latent tuberculosis: a multicentre cohort study and cost-effectiveness analysis. *Lancet Infect Dis* 2011; **11**(6): 435-44.

33. Pottie K, Lotfi T, Kilzar L, et al. The Effectiveness and Cost-Effectiveness of Screening for HIV in Migrants in the EU/EEA: A Systematic Review. *Int J Environ Res Public Health* 2018; **15**(8).

34. Hatzakis A, Wait S, Bruix J, et al. The state of hepatitis B and C in Europe: Report from the hepatitis B and C summit conference. *Journal of Viral Hepatitis* 2011; **18**: 1-16.

35. Mason LMK, Veldhuijzen IK, Duffell E, et al. Hepatitis B and C testing strategies in healthcare and community settings in the EU/EEA: A systematic review. *J Viral Hepat* 2019; **26**(12): 1431-53.

36. Hahne SJ, Veldhuijzen IK, Wiessing L, Lim TA, Salminen M, Laar M. Infection with hepatitis B and C virus in Europe: a systematic review of prevalence and cost-effectiveness of screening. *BMC Infect Dis* 2013; **13**: 181.

37. Myran DT, Morton R, Biggs B-A, et al. The effectiveness and cost-effectiveness of screening for and vaccination against hepatitis B virus among migrants in the EU/EEA: A systematic review. *International Journal of Environmental Research and Public Health* 2018; **15**(9): 1898.

38. Epidemiological assessment of hepatitis B and C among migrants in the EU/EEA; 2016.

39. Abubakar I, Stagg HR, Cohen T, et al. Controversies and unresolved issues in tuberculosis prevention and control: a low-burden-country perspective. *J Infect Dis* 2012; **205**: S293-300.

40. Dasgupta K, Menzies D. Cost-effectiveness of tuberculosis control strategies among immigrants and refugees. *Eur Respir J* 2005; **25**(6): 1107-16.

41. Bothamley GH, Ditiu L, Migliori GB, Lange C, contributors T. Active case finding of tuberculosis in Europe: a Tuberculosis Network European Trials Group (TBNET) survey. *Eur Respir J* 2008; **32**(4): 1023-30.

42. Coker R, Bell A, Pitman R, et al. Tuberculosis screening in migrants in selected European countries shows wide disparities. *Eur Respir J* 2006; **27**(4): 801-7.

43. Tuberculosis. London: National Institute for Health and Care Excellence; 2019 (NICE guideline NG33; <https://www.nice.org.uk/guidance/ng33>. 2019.

44. WHO. Systematic screening for active tuberculosis: principles and recommendations; 2013.

45. Korenev D. Regional Expert Group on Migration and Health in Eastern Europe and Central Asia (EECA). How much does it cost to treat HIV-positive migrants in Russia? Available from: <http://migrationhealth.group/en/how-much-does-it-cost-to-treat-hiv-positive-migrants-in-russia/> Accessed 14th July 2021. Press release reporting results of ES Zaiko, AA Popova, KA Barskiy, DS Kashnitskiy. Economic analysis of HIV care for foreign migrants in the Russian Federation. Regional Expert Group on Migration and Health [Russian]. Available from: <http://migrationhealth.group/wp-content/uploads/2021/05/Ekonomicheskoe-issledovanie.-Inostrantsy-s-VICH-final.pdf> Accessed 14th July 2021. 2021.

46. Miners AH, Martin NK, Ghosh A, Hickman M, Vickerman P. Assessing the cost-effectiveness of finding cases of hepatitis C infection in UK migrant populations and the value of further research. *J Viral Hepat* 2014; **21**(9): 616-23.

47. Campbell JR, Sasitharan T, Marra F. A Systematic Review of Studies Evaluating the Cost Utility of Screening High-Risk Populations for Latent Tuberculosis Infection. *Appl Health Econ Health Policy* 2015; **13**(4): 325-40.

48. Auguste P, Tsertsvadze A, Pink J, et al. Accurate diagnosis of latent tuberculosis in children, people who are immunocompromised or at risk from immunosuppression and recent arrivals from countries with a high incidence of tuberculosis: systematic review and economic evaluation. *Health Technol Assess* 2016; **20**(38): 1-678.

49. Nienhaus A, Schablon A, Costa JT, Diel R. Systematic review of cost and cost-effectiveness of different TB-screening strategies. *BMC Health Serv Res* 2011; **11**: 247.

50. Cost–effectiveness analysis of programmatic screening for latent tuberculosis infection in the EU/EEA. Stockholm: European Centre for Disease Prevention and Control; 2018 (ECDC Technical report; <https://ecdc.europa.eu/sites/portal/files/documents/LTBI%20cost-effectiveness%20report.pdf>, accessed 17 April 2021). 2018.

51. Programmatic management of latent tuberculosis infection in the European Union. Stockholm: European Centre for Disease Prevention and Control; 2018:49 (<https://www.ecdc.europa.eu/en/publications-data/programmatic-management-latent-tuberculosis-infection-european-union>, accessed 15 September 2021). *ECDC scientific advice* 2018: 49.

1. At the time of the search, the official country name for Türkiye was “Turkey”, which was the term used in our search strategy. [↑](#footnote-ref-1)
2. Defined as any individuals residing in a different country from the one in which they were born. [↑](#footnote-ref-2)
